# Evaluation of Border Entry Screening for Infectious Diseases in Humans

**DOI:** 10.3201/eid2102.131610

**Published:** 2015-02

**Authors:** Linda A. Selvey, Catarina Antão, Robert Hall

**Affiliations:** Curtin University, Perth, Western Australia, Australia (L.A. Selvey, C. Antão);; Monash University, Melbourne, Victoria, Australia (R. Hall)

**Keywords:** influenza, 2009 influenza pandemic, influenza A(H1N1)pdm09, humans, SARS virus, severe acute respiratory syndrome, quarantine, patient isolation, border crossing, border entry, mass screening, pandemic, disease transmission, infectious, communicable diseases, disease control strategies, viruses, health communication

## Abstract

In response to the severe acute respiratory syndrome (SARS) pandemic of 2003 and the influenza pandemic of 2009, many countries instituted border measures as a means of stopping or slowing the spread of disease. The measures, usually consisting of a combination of border entry/exit screening, quarantine, isolation, and communications, were resource intensive, and modeling and observational studies indicate that border screening is not effective at detecting infectious persons. Moreover, border screening has high opportunity costs, financially and in terms of the use of scarce public health staff resources during a time of high need. We discuss the border-screening experiences with SARS and influenza and propose an approach to decision-making for future pandemics. We conclude that outbreak-associated communications for travelers at border entry points, together with effective communication with clinicians and more effective disease control measures in the community, may be a more effective approach to the international control of communicable diseases.

## Abstract 2

Outbreak-associated communications for travelers and clinicians may be a more effective approach to the international control of communicable diseases.

Many countries instituted border screening in response to the severe acute respiratory syndrome (SARS) pandemic of 2003 and the influenza A(H1N1)pdm09 virus pandemic of 2009, and although not formally evaluated, the experiences of several countries have been documented (*1*–*11*). Given the recent emergence of the influenza A(H7N9) virus in many parts of China (*12*), Middle East respiratory syndrome coronavirus in Saudi Arabia (*13*), and the current, most widespread Ebola outbreak in Africa (*14*), it seems timely to consider the costs and the effectiveness of border screening, as shown by recent experiences. Herein, we discuss the use of border-screening measures instituted during the 2003 SARS pandemic and the 2009 influenza pandemic.

Border screening, together with isolation of persons identified with suspected cases of disease and quarantine of their contacts, is implemented to delay or prevent the entry of infected persons to a country/geographic area or to prevent the global spread of a disease from a source country. The intent of border screening is to detect possibly infectious persons at the border, either on entry to or exit from a country, so that they can be placed in isolation or prevented from traveling and spreading the disease elsewhere; however, this strategy is useful only if the intended goal is successfully achieved. Other potential benefits of border screening relate to increasing public awareness about and confidence in protection from the disease in question, but the scope of this article does not allow for a discussion of these benefits.

During the 2009 influenza A(H1N1)pdm09 virus pandemic, the World Health Organization advised persons who were ill with influenza to delay travel (*15*). Early during the SARS pandemic and in August 2014 during the Ebola virus epidemic, the World Health Organization recommended border exit screening of travelers from affected countries (*16*,*17*). Border screening can be undertaken through self-identification by means of health declaration cards, airline/transit agency notification to health authorities of sick passengers, visual inspection of travelers, and/or fever screening of travelers implemented through the use of infrared thermal image scanners (ITISs). Three key questions are the following: How effective have these measures been at detecting ill travelers? Are there situations in which border screening is likely to be effective? If border screening is not effective, are there any other measures that could be implemented to prevent the spread of disease beyond the source country? To explore these questions, we examined border-screening experiences during the influenza A(H1N1)pdm09 virus pandemic and the SARS pandemic. Questions relating to the effectiveness of border screening are relevant regardless of the situation in which they are applied, including limited screening from one part of the world or screening on isolated island countries, because the experiences relate to the effectiveness of the measure itself in detecting cases at the border.

## Border Screening and the Influenza A(H1N1)pdm09 Virus Pandemic

Because of a short incubation period and consequent short serial interval (i.e., time between the onset of the first case and the onset of subsequent case[s]), influenza virus causes explosive outbreaks despite its relatively low infectivity. Influenza A(H1N1)pdm09 virus, which spread rapidly throughout the world in 2009, was most likely established in Australia (*18*) and Japan (*19*) before border screening was initiated in those countries. Border screening to detect influenza-infected travelers is likely to be unsuccessful because persons with asymptomatic cases can be infectious, and fever is not a consistent symptom of influenza (*20*). This means that screening sensitivity is low and a substantial proportion of infectious persons will not be detected at the border, and those that are detected may well have transmitted the virus to other persons before being isolated. This was the experience of several countries during the influenza A(H1N1)pdm09 virus pandemic. For example, in Singapore, of the first 116 influenza A(H1N1)pdm09 virus–infected persons identified with a history of recent international travel, only 15 (12.9%) were identified through screening at the airport (*2*). In Japan, intensive border screening was in place at the main international airport during April 28–June18, 2009. Of 151 influenza cases that might have been acquired during travel overseas, only 10 (6.6%) were detected as a result of border screening in Japan (*4*). During the same period in New South Wales, Australia, an estimated 6.7% (3/45) of imported cases were detected at the border (*9*), and in Auckland, New Zealand, 5.8% (4/69) of the cases were detected at the airport (*10*). Singapore, Japan, and Australia, but not New Zealand, used ITISs to screen for fevers at their borders, even though the sensitivity of this screening was similarly low at the sites.

Before the influenza A(H1N1)pdm09 virus pandemic, a modeling study suggested that the use of thermal scanners at airports/entry points to screen incoming passengers or at exit points from countries where influenza virus is circulating could reduce the number of cases that would otherwise occur during a pandemic (*21*). However, the study assumed a 50% detection rate for all incoming infected persons, including those with asymptomatic cases and those incubating the virus (*21*). In practice, detection was substantially lower than that.

ITISs were used in many countries to detect febrile passengers. A review of hospital-based studies examining the efficacy of ITISs in detecting fever found that the sensitivity of fever detection ranged from 4% to 89.6%, and the positive predictive value with a 1% prevalence of fever ranged from 3.5% to 65.4% (*22*). A more recent study involving airline travelers estimated a positive predictive value of ITIS for fever detection of 0.9%–4.1% for detecting fever of any cause and a positive predictive value of fever for influenza of 2.0%–2.8% for detecting influenza-associated fever (*3*). Therefore, many persons with possible fever would have to be identified before a case of influenza was detected, and screening for fever is unlikely to be sensitive enough to detect sufficient numbers of influenza cases to prevent or slow the importation of a pandemic strain.

Several other models have assessed the role of travel restrictions on the international spread of influenza (*23*–*25*). These models concluded that unless travel restrictions prevented >99% of travel, they would, at best, delay the introduction of pandemic influenza by 2–3 weeks, and because of the explosive nature of the epidemic, would have no overall effect on the total number of cases (*23*–*25*). The results are effectively the same whether travel restrictions are used (as in these models) or screening and isolation/quarantine are used to limit the movement of possibly infectious persons. However, the conclusion from these models (i.e., that allowing only a small number of cases to enter a country would result in an epidemic of the same size as if travel restrictions were not in place) is applicable to screening. It is probable that entry screening with a low rate of detection of incoming cases would also be unlikely to significantly delay the commencement of an epidemic or reduce the total number of cases. The models had also not been validated using data from an influenza pandemic (*26*). Now that data from the influenza A(H1N1)pdm09 virus pandemic are available, there is an opportunity to validate the models examine the efficacy of border measures.

A substantial amount of resources were expended on border-screening measures in several countries, including Australia. At a time when clinical and public health services were stretched in responding to the pandemic, there were major opportunity costs resulting from the application of border screening (*4*,*9*). In New South Wales, it was estimated that the cost of staffing airport clinics was $50,000AUD/case detected (*9*).

## Border Screening and the SARS Pandemic

More than 10 years have elapsed since the SARS virus emerged in China. From its emergence in November 2002 through July 2003, the virus, which has an incubation period of 2–12 days (mean 4–5), infected >8,000 persons across 30 countries (*27*). Despite the lack of antimicrobial drugs or a vaccine, the epidemic was controlled worldwide through a combination of early isolation of case-patients, quarantine of contacts, and strict infection control measures (*20*,*27*).

Fraser et al. (*20*) modeled the control of communicable diseases according to the diseases’ characteristics of infectiousness during the incubation period and in asymptomatic infections. According to this model, public health measures are likely to be effective if persons are infectious only when symptomatic, particularly if infectivity peaks after the onset of symptoms. This means that infected persons are not infectious during the incubation period or during asymptomatic infection. During SARS virus infection, peak viremia (and assumed infectivity) occurs 10 days after symptom onset (*28*), and this timing coincides with the severity of symptoms. Persons with asymptomatic infection and persons in the incubation period do not appear to be infectious (*27*,*28*). Therefore if case-patients are isolated within 2–3 days of infection, transmission will be limited (*29*). If contacts are quarantined until beyond the incubation period, this will also limit further transmission. High fever (>38°C) is a common symptom among persons seeking medical care for SARS, but case-patients with fever of <38°C or who were afebrile have been described and have been implicated in the transmission of SARS in a health care setting (*30*–*33*). This information suggests that active case finding, isolation, strict infection control, and contact tracing will limit the spread of SARS, and modeling suggests that the combination of these measures would be the most effective control strategy (*20*,*29*). This information also suggests that border measures that involve effective case detection (i.e., a high proportion of cases detected), especially if associated with opportunities for effective contact tracing (i.e., contacts quarantined within 2 days of case-patient contact), could be useful strategies for delaying the entry of SARS into a country and limiting opportunities for the virus to spread. However, the long SARS incubation period means that cases of imported disease could easily occur through the border entry of infectious, asymptomatic persons.

During the SARS epidemic, several countries instituted border measures, including travel warnings, educational information for travelers, and border screening. In Australia, Canada, and Singapore, a combination of border screening measures was instituted, yet no confirmed SARS cases were detected in any of the 3 countries (*5*–*7*). In Australia, where ITISs were not used, 4 suspected/probable SARS cases were detected at the border. Those 4 cases represented 13.8% of the 29 persons detected in Australia with suspected/probable SARS during the screening period who were symptomatic at the time of arrival in the country (*5*). Five suspected/probable SARS case-patients arrived in Canada during the screening period; symptoms developed in all 5 patients after arrival, and none of the cases were detected at the border (*6*). The authors concluded that because of the very low prevalence of infection among travelers, the positive predictive value of any border screening would be effectively zero (*6*).

Two independent modeling studies (*29*,*34*) modeled the effect of entry screening for SARS on SARS importation and subsequent spread. Glass and Becker (*29*) concluded that entry screening for SARS would not reduce the probability of an outbreak of 100 cases by >7%; this conclusion is based on the assumption of screening effectiveness equivalent to that estimated based on the Australian experience. Goubar et al. (*34*) also concluded that entry screening would play a minimal role in reducing the number of imported cases, on the basis that border screening would miss infected travelers who are currently incubating the infection (*34*). Both studies concluded that SARS transmission within a country could be more effectively limited by gearing-up health services to enable early detection and isolation of case-patients than by investing in border screening (*29*,*34*).

## To Screen or Not To Screen

We do not recommend border screening at any time during the evolution of an influenza pandemic because the sensitivity and specificity of influenza screening are low, regardless of the method (e.g., self-identification, thermal scanning, and/or visual inspection). Border screening is resource intensive, and there is a significant opportunity cost for other public health measures if border screening is in place. For example, in Australia during May 2009 (i.e., during the influenza pandemic), an average of 28,685 persons arrived at 8 airports via international air flights (*1*). Entry screening was in place at the time, and each screening point with an ITIS required 1–2 operators at all times when flights were arriving. Trained nurses were required to be present at each airport at all times when there were incoming flights to provide follow-up for any passengers identified through ITIS screening or who self-identified as being unwell. An additional person was employed at each airport at all times when flights were arriving to assist with administrative activities. During April 28, 2009–June 1, 2009, a total of 15,457 (≈1.5%) airline travelers arriving at airports across Australia were identified as being unwell. Most (84%) of these persons self-identified as being unwell on health declaration cards; only 0.5% were identified by the use of an ITIS (*1*). Of these 15,457 persons, only 154 were subsequently treated as if they were infected with the pandemic influenza virus.

Influenza outbreaks are difficult to control without the use of vaccines and antiviral drugs. The public health response should focus on early identification and treatment of cases at risk of becoming severe; social-distancing measures applied at the community level; infection control measures; vaccination (when a vaccine becomes available); and in some cases, antiviral prophylaxis. Focusing on these measures instead of border screening will be more fruitful.

Compared with influenza, SARS is more amenable to border screening because fever is a more consistent symptom and infected persons are not infectious when asymptomatic or during the incubation period. However, persons who are incubating the SARS virus will not be detected by screening, and, given the low prevalence of infection even in source countries, the positive predictive value of screening will be very low. Therefore, we also do not recommend border screening for SARS. SARS is, however, amenable to control through the use of a combination of measures: early isolation of confirmed case-patients, quarantine of case-patient contacts, and strict infection control (*20*,*27*). Measures that will enable the early detection and isolation of case-patients and quarantine of contacts should be the focus of resource allocation.

## Communication as a Border Measure

Communication with incoming travelers was a key component of border activity during the SARS and influenza A(H1N1)pdm09 virus pandemics and during other disease outbreaks (*8*,*35*,*36*). Communication can take many forms, including informational videos, posters, signs, in-flight announcements, flyers, and health alert notices (HANs) (*36*). During the 2009 influenza pandemic, 44% and 84% of travelers identified as unwell on arrival in Singapore (*2*) and Australia (*1*), respectively, self-identified as being ill; this finding suggests that communication to incoming travelers can be a useful mechanism to encourage self-reporting. However, the evidence of the effectiveness of communication measures at borders is limited (*6*,*36*). Travel HANS (T-HANs) have been used in the United States by the Centers for Disease Control and Prevention (Atlanta, GA) since the 1970s as a communication tool directed to incoming travelers. T-HANs provide travelers with information about a current disease outbreak, symptoms of the disease, and advice about seeking medical care should symptoms occur. T-HANs also include clinical guidance and resources for physicians. Selent et al. (*35*) evaluated the effectiveness of T-HANs in encouraging the self-identification and health care–seeking behavior of incoming travelers from Haiti during the cholera epidemic in that country in 2010. The evaluation suggested that the T-HANs provided a small positive influence on health care–seeking behavior among incoming travelers (*35*). The use of current communication technologies (e.g., the Internet or short text messages to mobile phones) could also be investigated. SMS (short message service) messages, for example, have been used successfully in other areas of public health (*37*).

The use of T-HANs and other communication methods is a potentially worthwhile border measure that could assist with the early identification and appropriate management of incoming passengers with a disease of interest. Such measures need to be accompanied by the provision of appropriate health care for travelers who are deemed ill, and must be easily understandable. As with any health communication endeavor, effective communication requires multiple modes of communication and tailored messages (*38*).

Although the provision of consistent and repeated early warnings and information about infectious disease outbreaks to local clinicians is not a border measure, it can be highly effective in supporting the rapid recognition and isolation of possibly infectious incoming travelers. This fact is exemplified by the experience of SARS in Canada, where alert clinicians in Vancouver, British Columbia, isolated a patient with SARS within 15 minutes of his/her arrival at the clinic and used appropriate respiratory protection, but clinicians in Toronto, Ontario, did not quickly isolate a patient with SARS or use adequate respiratory protection when treating the patient. Both case-patients sought care at a hospital on the same day during a time when significant SARS transmission was ongoing in Ontario but not in Vancouver. Subsequent investigation identified well-communicated and repeated warnings about SARS to local clinicians as being an important factor in limiting further spread of SARS in Vancouver (*39*).

## Conclusions

Historically, most attempts at border screening have been ineffectual, as demonstrated by the pandemic spread of SARS and influenza A(H1N1)pdm09 to many countries despite the use of border screening. Modeling and observational studies have indicated that border screening is likely to be unsuccessful in preventing or delaying the entry of such diseases into a country. Border screening generally has high opportunity costs, both financially and in terms of the use of scarce public health staff resources at a time of high need. We conclude that border screening should not be used. Instead, the less costly measure of providing information to arriving travelers is recommended, together with effective communication with local clinicians and more effective disease control measures in the community.
